# Subjective well-being predicts Covid-19 risk in the elderly: a case–control study

**DOI:** 10.1186/s12877-022-03614-2

**Published:** 2022-11-22

**Authors:** Fatemeh Kashefi, Afsaneh Bakhtiari, Hemmat Gholinia, Fatemeh Bakouei, Mahbobeh Faramarzi

**Affiliations:** 1 Family Health Unit of Fereydoun-Kenar Health Network, Mazandaran University of Medical Sciences, Sari, Iran; 2 Social Determinants of Health Research Center, Babol University of Medical Sciences, Babol, Iran; 3 Health Research Institute, Babol University of Medical Sciences, Babol, Iran; 4 Infertility and Reproductive Health Research Center, Health Research Institute, Faculty of Nursing & Midwifery, Babol University of Medical Sciences, Babol, Iran; 5 Social Determinants of Health Research Center, Health Research Institute, Babol University of Medical Sciences, Babol, Iran

**Keywords:** Subjective well-being, Coronavirus, Elderly

## Abstract

**Background:**

Covid-19 is a serious public health concern. Previous studies have shown that although there are concerns about the subjective well-being (SWB) of older people in the Covid period, the link between SWB and the risk of Covid-19 is still unclear. This study aimed to investigate the predictive effect of SWB on the Covid-19 risk in the elderly as well as the determinants of SWB.

**Methods:**

This case–control study was performed in the elderly over 60 years of age. The case group consisted of all hospitalized patients with COVID-19 and the control group from the same population with no history of COVID-19 matched by age, sex, and place of residence. Data collection tools included a demographic questionnaire and SWB scale of Keyes and Magyarmo to measure emotional, psychological and social well-being. All data were analyzed via SPSS and STATA software. Multiple binary logistic regression was run to predict the probability of Covid-19 risk on the values of total SWB and its three subscales and multiple linear regression to identify SWB determinants.

**Results:**

The results showed that increasing one unit in total SWB reduces the risk of Covid-19 by 4% (OR = 0.969, CI = 0.947–0.991, *p* = 0.006). Emotional well-being with 0.823 had the highest odds ratio for predicting Covid-19 risk, followed by social well-being with an odds ratio of 0.981. Increasing age and education, better economic status, marriage against celibacy, lack of comorbidity, and a better understanding of own health were associated with greater SWB.

**Discussion:**

This study provides evidence for the protective effect of SWB on Covid-19 risk. To promote SWB, we need to focus on the elderly with higher financial worries and comorbidities, as well as those with less education, health perception and SWB. Therefore, it will be important for the elderly to determine strategies to improve SWB during the epidemic.

**Supplementary Information:**

The online version contains supplementary material available at 10.1186/s12877-022-03614-2.

## Background

Subjective well-being (SWB) has emerged as an important concept in health research and measures in recent decades along with the term successful aging. SWB is defined as a personal perception and experience of the proper balance of positive and negative emotions, cognitive and emotional assessments of one's life, and life satisfaction [1]. SWB is an individual assessment of the quality of life (QoL) so it is convergent with the definition of QoL [2]. There are two main types of well-being concepts, i.e., hedonic and eudemonic. Hedonic well-being refers to the emotional aspects of positive psychology, such as happiness, enjoyment of life, comfort, and the assessment of well-being is related to life satisfaction, while Eudemonic well-being focuses on the elements of a good and valuable life, such as purpose, growth, and meaning of life [3]. Well-being is a dynamic concept that includes emotional, psychological and social subscales. Emotional well-being is the ability to create positive emotions, moods, thoughts and feelings, and adaptability in the face of difficult and stressful situations. As an individual concept, psychological well-being addresses the challenges that adults face in their private lives, and social well-being represents a general concept that focuses on the social tasks that adults face in their social structures; it shows whether people are doing well in their social world [4].

Older age does not necessarily increase psychological vulnerability. Although aging is associated with mitigated performance, cognition, health, and social interactions, a high degree of stability (or increase) in SWB has been consistently observed at higher ages —a phenomenon called the"well-being paradox" and/or "stability despite loss" [5, 6]. Reports suggest that SWB changes from the young to middle and older ages. There is a U-shape relationship between well-being and age indicating that well-being is compromised in middle ages and enhanced at both ends of age, i.e. in the young and old ages [3]. In line this concept, the selective emotional-social theory asserts at higher ages, the improved emotional wisdom leads to a wiser selection of more satisfying events, friendships, and experiences [3]. Thus, despite events such as the death of a loved one, retirement, deteriorating health, and declining income (although financial needs may also decrease), older people maintain and even increase their well-being by focusing on a more limited set of contacts and social experiences [7]. Therefore, although older populations are generally less healthy and less productive, they may experience more life satisfaction than middle-aged people and experience less stress, anxiety, and anger [8].

Well-being and health have a strong, two-way relationship, which can become more important at older ages, simply because there is a higher prevalence of chronic diseases in the aged population [1]. With improved life expectancy and more effective treatments for life-threatening diseases, well-being gains more momentum in older ages [5]. Researches show that SWB may even be a protective factor for the health of the elderly, reduce the risk of chronic physical diseases, such as cardiovascular disease (CVD), diabetes, brain accidents, stress and depression, cancers [9], chronic lung disease [10] and osteoarthritis [9], and increase life expectancy [11]. There is ample evidence that greater SWB, especially emotional well-being, is associated with reduced mortality in prospective epidemiological cohort studies [12, 13] and meta-analyses [14, 15].

Covid-19 is a serious public health concern, and the elderly are particularly vulnerable to severe health consequences [16–18]. Reactions to COVID-19 have varied during the crisis; for some people, Covid-19 has imposed restrictions, while others have relied on guidelines and recommendations to slow the spread of the disease. However, its impact on daily life, especially for people over 70, has been enormous [16]. Arbitrary age restrictions and quarantines may also put more pressure on older people [19]. Statistics in Iran indicate more mortality and morbidity in the elderly during this period [20]. The World Health Organization has warned of reduced well-being during the epidemic, especially among the elderly [5]. In the United States, concern has risen and well-being has been at its lowest level in the past 12 years [18]. Many older people do not have the resources to cope with COVID-19 stress. This may include material resources (e.g., lack of access to smart technology), social resources (e.g., few family members or friends), and cognitive or biological resources (e.g., inability to exercise or participate in routine activities/programs) [21].

Research on SWB and the consequences of various diseases caused by it, especially in older ages, is in its infancy. SWB may act as a preventative factor in health. Well-being is especially important in the elderly and is related to their QoL. The relationship between SWB as an independent variable and Covid-19 risk as a dependent variable has not been investigated. The results of this study can encourage health care systems to address positive psychological states in addition to disease and disability. This study has mainly focused on the predictive effect of total SWB and its subscales on Covid-19 risk, and has aimed to examine the determinants of SWB in the elderly as a secondary purpose.

## Methods

### Study design and participants

This case–control study was conducted from April 20 to September 21, 2020, to compare the emotional, psychological and social well-being of the elderly in the two groups with and without Covid -19 in the Fereydoun-Kenar city located in Mazandaran province, north of Iran. This study consisted of 180 community-dwelling adults in the two groups of the case (*n* = 90) and control (*n* = 90). The STROBE Checklist was followed for observational studies.

### Study population

Eligibility criteria include age 60 years and older, consent to participate in the study, ability to speak Persian, lack of Covid-19 in the acute phase, no history of cognitive impairment (mini-mental state score ≤ 23), acute or severe diseases, unpleasant events in the last three months, and no/mild limitation in daily activities (i.e., able to eat, toilet, dress, bathe/shower without difficulty, likely to have some difficulty getting in/out of bed/chairs and/or walking).

### Case–control selection

The statistical population in both case and control groups was identified from the registration systems of the family health unit of the Fereydoun-Kenar health network, a population base that covers all elderly people living in the city. Throat samples were obtained from all susceptible people (those with fever, cough, and shortness of breath). Then, the prepared samples were examined by the reverse transcription-polymerase chain reaction (RT-PCR) test. If the test result was positive, the person was considered a case, otherwise, they were regarded as a control. The case group was all hospitalized patients with COVID-19. Thus, out of 117 hospitalized patients during the study period, 27 were excluded because they did not meet the eligibility criteria, so 90 patients were included in the study. A three-month period was considered to minimize the impact of hospitalization on SWB in the case group. For each case, an elderly person from the population covered by the same center was randomly selected who matched in terms of gender, age (± 3 years) and place of residence, with no history of Covid-19.

### Exposure measurement: total SWB, emotional, psychological and social well-being

The SWB scale of Keyes and Magyarmoe was consulted to measure the outcome. It was devised in 2003 to measure emotional, psychological, and social well-being during the last month [22]. It consists of 45 questions. The first 12 questions are related to emotional well-being in the two components of positive and negative emotions, each with 6 questions. The sum of the scores of these two components shows the total score of emotional well-being. The next 18 questions are related to psychological well-being with the six components of personal growth, positive relations with others, autonomy, environmental mastery, purpose in life, and self-acceptance. Finally, the next 15 questions are related to social well-being with social integration, social contribution, social coherence, social acceptance, and social actualization components. The answers were scored based on the Likert scale. The minimum and maximum scores in each subscale and the total well-being scale are summarized in Table 1.

**Table 1:** Scoring the total subjective well-being and its subscales

Well-being components	Minimum score	Maximum score
Emotional well-being	12	60
Psychological well-being	18	126
Social well-being	15	105
Total subjective well-being	45	291

This questionnaire does not have a cut-off point. A higher score on all three subscales as well as on the total scale indicates better SWB. This means that people with greater SWB experience more positive emotions. They have a positive evaluation of the events around them and describe them as pleasant. These people have a better sense of control over life issues and their success rate and satisfaction with life is higher while people with a low sense of well-being evaluate their life events and situations as unfavorable. This questionnaire was implemented and validated by Golestanibakht (2007) on 57 subjects and the correlation coefficient of the questionnaire was 0.78 [23]. Also, its sub-scales, including emotional, psychological, and social well-being were reported to be 0.76, 0.64, and 0.76, respectively. Based on Cronbach's alpha, the internal consistency coefficient for the whole questionnaire was 0.80 and for its subscales, it was 0.86, 0.80 and 0.61, respectively [23]. Due to maintaining social distance, the questionnaire was completed by telephone interview by a member of the research team. Telephone interviews were conducted in the morning when the respondents' mental and physical condition was most favorable. Before starting the study, the participants were informed of the purpose of the study and were taught how to answer the questions. During the survey, anyone could receive additional information in case of ambiguity. Each interview lasted from 60 to 75 min. The participants' responses and statements were carefully recorded.

### SWB determinants

The determinants of SWB included in the multivariate linear regression model were age, gender, occupation, education, economic status, marital status, living status, self-rated healthy and comorbidity, which were completed through a questionnaire.

### Ethical considerations

The ethics committee of Babol University of Medical Sciences (BUMS) approved this study before starting the formal survey (ethical code: IR.MUBABOL.REC.1399.262). All participants signed the informed consent form and were given the chance to withdraw from the study at any stage. The Helsinki Declaration principles were observed throughout the study.

### Statistical analyses

All data were analyzed via SPSS v. 23.0 (SPSS Inc., Chicago, Illinois, USA) and STATA v. 16 software. Multivariate binary logistic regression was employed to predict the probability of a change in the classified dependent variable (Covid-19 risk, yes/no), conditional on the values of independent variables (mainly total SWB and its three subscales and personal variables as associated covariates). In addition to supplying an estimate of conditioned probability, the model allows one to assess the degree of the effect of the selected independent variables on the occurrence of the dependent variable. Multivariate linear regression was performed to identify the determinant of the total SWB as the secondary purpose. An independent t-test was applied to compare the mean of total SWB, the subscales and their components in the two groups. Χ^2^ test was run to compare the frequency of demographic characteristics classified into the two groups. *p* < 0.05 was statistically significant.

## Results

The mean age was 68.2 ± 6.8 years (range 60 to 86 years). Of these, 78 (43.3%) were women and 102 (56.7%) were men with equal distribution in the two groups. The characteristics of the participants are shown in Table 2. The χ2 test showed a statistically significant difference between the two groups in terms of the infection of other family members with Covid-19, comorbidity and self-rated health. Regarding the observance of the health protocols, the majority of participants in both groups (90%) reported a 30-s washing of their hands and social distancing, while the use of the mask was reported by only 30% of the case group versus 55.6% in the control group (*p* = 0.001). Also, the most common reason for leaving home during quarantine was shopping for necessities (42.8%), meeting the health team (20%), and business activities (9.4%).

**Table 2:** Personal characteristics of the participants in the groups

Variables	All (*N* = 180)	Case (*N* = 90)	Control (*N* = 90)	*p*.value
**Occupation**				0. 420
Retired	33 (18.3)	18 (20.0)	15 (16.7)	
Business person	56 (31.1)	30 (33.3)	26 (28.9)	
Unemployed	16 (8.9)	6 (6.7)	10 (11.1)	
Housewife	75 (41.7)	36 (40.0)	39 (43.3)	
**Educational level**				0.123
Literacy	89 (49.7)	38 (42.2)	51 (57.3)	
< Diploma	72 (40.2)	40 (44.4)	32 (36.0)	
Diploma	16 (8.9)	10 (11.1)	6 (6.7)	
University	2 (1.1)	2 (2.2)	0. (0.0)	
**Income adequacy from the individual perspective**				0.765
Enough	31 (17.4)	17 (18.9)	14 (15.9)	
Nearly enough	108 (60.7)	55 (61.1)	53 (60.2)	
Not enough	39 (21.9)	18 (20.0)	21 (23.9)	
**Marital status**				0.500
Married	153 (85.0)	77 (85.6)	76 (84.4)	
Single	27 (15.0)	13 (14.4)	14 (15.6)	
**Chronic diseases**				0.185
No	93 (51.7)	43 (47.8)	50 (55.6)	
Yes	87 (48.3)	47 (52.2)	40 (44.4)	
**Comorbidity**				0.001
No	158 (87.8)	74 (82.2)	84 (93.3)	
Yes	22 (12.2)	16 (17.8)	6 (6.7)	
**Self-rated healthy**				0.001
Not healthy	49 (27.2)	35 (38.9)	14 (15.6)	
Like others	67 (37.2)	36 (40)	31 (34.4)	
Better than others	64 (35.6)	19 (21.1)	45 (50)	
**Living status**				0.170
Alone	16 (8.9)	6 (6.7)	10 (11.2)	
Living with family (spouse and children)	58 (32.4)	34 (37.8)	24 (27.0)	
Living with spouse	82 (458)	36 (40.0)	46 (51.7)	
Living with children	23 (12.8)	14 (15.6)	9 (10.1)	
**Infection of other family members with covid-19**				0.001
No	108 (62.8)	38 (43.2)	70 (83.3)	
Yes	64 (37.2)	50 (56.8)	14 (16.7)	

Values are number (percentage)

Total SWB in the elderly with Covid-19 was significantly lower than the elderly without it (*p* = 0.001). The relationship with the SWB subscales also revealed a significant decrease in the emotional well-being subscale (*p* = 0.001) and its positive emotion component (*p* = 0.002) and a significant increase in the negative emotion component in the case group compared to the control group (*p* = 0.001) (Tale 3). The psychological and social subscales did not show a significant difference between the two groups. However, the components of social cohesion and social realization of the social well-being subscale were significantly lower in the case group than in the control group (*P* = 0.036, 0.001, respectively).

Emotional well-being with 0.823 has the highest odds ratio for predicting infection with Covid-19, followed by social well-being with an odds ratio of 0.934. This means that a 1-unit increase in emotional and social well-being reduces 0.18% and 0.07% chance of infection with Covid-19, respectively (Table 4). Cox and Snell R2 and Nagelkerke R2 indicated that 25% and 33% of the variation in the dependent variable is explained by the logistic model, respectively. We ran this model once again for total SWB instead of its subscales. The results showed that increasing one unit in total SWB reduces the risk of Covid-19 by 4% (OR = 0.969, CI = 0.947–0.991, *p* = 0.006). The Cox & Snell R2 and Nagelkerke R2 were 0.11 and 0.15. This means that a combination of the introduced independent variables accounts for 11–15% of infections with Covid-19 variance.

Table 5 shows the associations between the determinants and total SWB and its subscales in the elderly by linear multiple regression analysis. The results indicated that with age, negative emotion increases to a small but significant amount (0.083, *p* = 0.031). Significant improvement in psychological well-being was also observed with age (0.336, *p* = 0.005). However, age did not show a significant relationship with other SWB subscales. Also, advancement in education from illiteracy to diploma and above was significantly associated with lower total SWB score and all three subscales. Improving the economic status from insufficient to almost sufficient was accompanied with a sevenfold increase in total SWB (*p* = 0.020), threefold in emotional well-being (*p* = 0.011), sixfold in psychological (0.006), and 4.5-fold in social well-being (*p* = 0.011). In sufficient economic conditions, this increase reached 18, 9, 16 and 11.5 times, respectively. Marriage versus singleness was associated with a 4.6-fold increase in emotional well-being (*p* = 0.002) and a threefold increase in positive emotion (*p* = 0.002). Nevertheless, negative emotion was reduced by almost twice (*p* = 0.029). Lack of comorbidity was associated with an eightfold improvement in total SWB (*p* = 0.001), a sixfold augmentation in psychological well-being (*p* = 0.001), and a threefold increase in social well-being (*p* = 0.014). Finally, older people who self-reported better health than others showed a tenfold improvement in total SWB (*p* = 0.001), a sixfold elevation in the emotional subscale (*p* = 0.001), a ninefold augmentation in the psychological subscale (*p* = 0.001), and a sixfold increase in the social subscale (*p* = 0.001), compared to those who reported their health worse than others. However, marital status and gender did not show any significant relationship with total SWB and its subscales.

The R-squared for the total SWB variable was 0.400, which means that a combination of introduced explanatory variables, accounts for 40% of poor total SWB variance. This value was 0.378, 0.456 and 0.339 for emotional, psychological and social well-being, respectively (Table 5).

## Discussion

This case–control study investigated the predictive effect of SWB on COVID-19 risk in an Iranian sample of the elderly. Furthermore, the factors affecting SWB in the aging context were examined by multiple linear regression. The analysis revealed that total SWB and its subscales, including emotional and social well-being, can predict the chance of developing Covid-19 in the elderly. Previous studies have shown that there are concerns about the well-being of older people in the Covid period [1, 2], but the link between SWB and Covid-19 risk is still unknown. As well-being is of primal status in the elderly, evidence suggests that positive hedonic states, life evaluation, and eudemonic well-being are associated with enhanced health and QoL as people age [3]. The following discussion describes this finding (Tables 3 and 4).

**Table 3:** Subjective well-being of the elderly with and without coronavirus

Well-being Components	Case (*N* = 90)	Control (*N* = 90)	95% CI	*p*.value
**Emotional**	35.57 ± 8.04	39.84 ± 5.43	-6.29. -2.26	0.001
Positive emotions	13.61 ± 4.27	16.18 ± 3.90	-3.77, -1.36	0.002
Negative emotions	23.67 ± 2.66	21.96 ± 4.48	-2..79, -0.625	0.001
**Psychological**	62.38 ± 14.93	62.11 ± 10.57	-3.539, 4.072	0.890
Self-acceptance	9.41 ± 3.86	9.26 ± 2.61	-0.814, 1.125	0.752
Purpose in life	11.71 ± 2,42	11.80 ± 2.35	-0.791, 0.614	0.803
Environmental mastery	10.01 ± 3.64	10.22 ± 3.06	-1.20, 0.78	0.675
Positive relations with others	8.63 ± 3.44	8.58 ± 2.44	-0.824, 0.935	0.901
Personal growth	11.73 ± 3.93	11.04 ± 3.21	-0.368, 1.746	0.200
Autonomy	10.88 ± 3.83	11.21 ± 1.97	-1.23, 0.563	0.464
**Social**	51.66 ± 11.50	54.02 ± 7.30	-5.201, 0.468	0.101
Social coherence	10.43 ± 4.12	11.58 ± 3.05	-2.212, -0.77	0.036
Social integration	9.64 ± 4.25	10.32 ± 2.63	-1.718, 0.362	0.200
Social acceptance	11.93 ± 2.61	11.48 ± 2.52	-0.301, 1.212	0.236
Social contribution	8.10 ± 2.31	7.56 ± 2.06	-0.100, 1.189	0.097
Social actualization	11.54 ± 3.10	13.09 ± 1.98	-2.311, -0.778	0.001
**Total subjective well-being**	149.60 ± 20.23	155.98 ± 13.71	-11.468, -1.288	0.014

Independent T-test

**Table 4:** Correlation of Covid-19 infection status with total subjective well-being and its subscales in elderly people

Variables	B	S.E	*p*-value	OR (95%CI)
Emotional well-being	-0.195	0.039	0.001	0.823 (0.762–0.889)
Psychological well-being	-0.019	0.024	0.426	0.981 (0.937–1.028)
Social well-being	-0.068	0.027	0.013	0.934 (0.886–0.986)
Cox & Snell R Square	0.252			
Nagelkerke R Square	0.337			
Total subjective well-being	-.032	0.012	0.006	0.969 (0.947–0.991)
Cox & Snell R Square	0.108			
Nagelkerke R Square	0.145			

Multivariate binary logistic regression by SPSS

Adjusted for age, gender, occupation, education, economic status, marital status, comorbidity and living status

Research on the relationship between SWB and the repercussions of other diseases has remained relatively new and often limited to chronic diseases such as CVD, diabetes, and hypertension. A longitudinal study with a 10-year follow-up on the elderly to assess disability and chronic disease–free life expectancy showed that higher SWB at older ages was associated with a longer, healthier life. In older ages, individuals experience greater enjoyment of life, have no depressive symptoms, and are more likely to remain in good health during the following decades, free from disability or serious chronic health conditions [1]. Other studies also documented an association between SWB and coronary heart disease [24], arthritis [25], frailty [26], metabolic syndrome [27] and respiratory infections [9].

The researches on SWB and health have identified two distinct perspectives in this field [9]. Greater SWB is associated with lower rates of cancer and breast cancer in particular [28, 29], Type 2 diabetes [30] and CVD [24]. However, there is conflicting evidence and other studies have reported no association between SWB and breast cancer [31] or heart disease [28]. The question remains as to whether the relationship between SWB and disease risk is similar across diseases. Many chronic diseases have several common risk factors, but it is not clear whether chronic diseases share another risk factor in the form of SWB. Some researchers [9, 28] have suggested that SWB may provide a "broad base of resilience" to chronic diseases. However, others [1, 13, 18] argued that because diseases might have different physiological processes and causes, the strength of a relationship between SWB and disease risk varies across diseases most notably with some diseases that have little or no connection with previous SWB.

On the other hand, the effect of SWB may be mediated by intermediates of physiological systems. For instance, SWB improves healthy life expectancy through two broad sets of mechanisms. Firstly, greater SWB is associated with optimal lifestyle choices, including more physical activity, less smoking, better sleep, and safer use of preventative health care [1, 14]. Healthier lifestyles, in turn, may delay disability as well as reduce the chronic physical illness risk. Secondly, SWB is associated with a range of biological processes, including decreased cortisol output, lower inflammatory cytokines concentrations, and higher levels of serum antioxidants [32, 33]. These processes protect against an increased risk of disability and coronary heart disease, diabetes, and other serious health conditions [9]. SWB, on the other hand, may act directly by influencing physiological processes associated with diabetes risk. For example, an increase in C-reactive protein (CRP) previously associated with low SWB [32] is a strong independent predictor of Type 2 diabetes [30]. SWB may also influence the risk of chronic lung disease by being associated with an inflammatory response, similar to arthritis [9].

Our study showed that lower SWB could be a predictor of Covid-19 risk. Although the mechanism of this relationship is unclear, based on the available literature, it can be assumed that well-being may affect the risk of infection through three possible pathways: 1. direct impact on neurobiological pathways, 2. indirect effects through health behaviors & life style, and 3. promoting psychosocial resources to protect against stressful events [34]. SWB reflects all the conditions that enable our nervous system to integrate and translate them into a language that the immune system can read. SWB may be as a safety signal that changes the immune system's priorities depending on social status, health, safety or nutritional status [35]. The research has shown that lonely individuals present the most active antibacterial and proinflammatory genetic pathways, while pathways that promote antiviral responses are preferred by individuals who do not feel lonely. This, in turn, can be interpreted as an adaptation to living conditions with a greater likelihood of injury and subsequent bacterial and viral infection due to lack of external support or increased risk of viral infection due to repeated contact with other people [36]. A recent study is consistent with these data and explained a physiological association with elevated levels of proinflammatory cytokines interleukin (IL) -6 and tumor necrosis factor (TNF) -a during the inflammatory immune response to experimental endotoxemia in individuals who reported feelings of social disconnection [10]. Current approaches often use traditional Oriental meditation practice programs such as yoga, tai chi, and qui gong or other relaxation techniques, with anti-inflammatory results mainly such as lowering the plasma level of clinical inflammatory markers, CRP, or IL-6 [34].

Similarly, people with greater SWB tend to self-report infection control behaviors during the Covid-19 period (such as face covering, social distancing isolating, putting packages and shopping aside, cleaning and disinfecting, and hand washing) and healthy lifestyle behaviors [36]. Regarding psychosocial mechanisms, studies have shown that positive personality traits, such as optimism, mindfulness, and resilience may protect against the negative mental health consequences of COVID-19 fear [5, 16, 18]. Optimism is associated with a variety of adjustment outcomes such as improving SWB, physical health, and coping with uncontrollable life events [9]. In addition, being aware of own experiences and accepting negative thoughts and feelings is associated with a reduction in psychological distress during stressful life events [35]. Finally, resilience, meaning the ability to recover from stress can reduce the negative impact of traumatic life events on mental health [5].

Determinants of SWB in the context of aging in the present study were education, economic status, occupation, comorbidity and self-rated healthy obtained from multiple linear regression analysis (Table 5). It has been shown that comorbidity and perceived health plays an important mediating role between physical health and SWB [36–38]. Also, recent studies demonstrated that variables such as socioeconomic status are strong determinants of SWB in older ages [24, 35]. Meanwhile, employment is one of the important indicators of active aging. With increasing age, the variety in daily activities decreases and people spend more time in passive leisure activities. A daily routine without meaningful activities and with a low activity level can lead to a decrease in physical or cognitive functions in the elderly population [39]. Participation in social activities is very important for the personal well-being and QoL of the elderly over 65 years of age. When people participate in diverse occupations, they can achieve a stable and harmonious situation in life, which has a positive effect on reducing stress and maintaining their health, in addition to income [40]. Therefore, elderly people are advised to discover new activities to help them spend as much active time as possible.

**Table 5:** Correlation of the determinants with total subjective well-being and its subscales in elderly people

Determinants	Total subjective well-being	Emotional well-being	Psychological well-being	Social well-being
**Group** (unhealthy vs. healthy)	-6.107^a^ 0.005^b^ -10.355, -7.860^c^	-4.894 0.001 -6.664, -3.125	0.794 0.599 -2.182, 3.771	-2.007 0.109 -4.470, 0.454
**Age** Mean (SD)	0.328 0.054 -0.006, 0.663	0.032 0.646 -0.107, 0.172	0.336 0.005 0.101, 0.571	-0.040 0.684 -0.234, 0.154
**Gender** (female vs. male)	0.477 0.852 -4.577, 5.533	-0.534 0.617 -2.640, 1.572	0.030 0.986 -3.513, 3.574	0.981 0.510 -1.949, 3.912
**Occupation** **(**employed vs. unemployed)	6.469 0.022 0.956, 3.983	0.878 0.451 -1.418, 3.175	3.062 0.120 -0.802, 6.927	2.529 0.120 -0.667, 0.725
**Education** (vs. Illiterate**)** ** < **Diploma	-4.129 0.309 -10.122, 3.863	-2.216 0.030 -5.877, -4.131	-5.598 0.050 -11.201, 0.005	-2.480 0.292 -7.114, 2.153
≥ Diploma	-6.717 0.006 -4.501, -1.934	-3.949 0.020 -3.100, -0.492	-5.374 0.002 -8.727, -2.020	-3.559 0.012 -6.333, -0.786
**Economic status** (vs. inadequate) Almost enough	6.957 0.020 1.330, 3.110	3.186 0.011 2.623, 5.748	5.727 0.006 1.626, 9.829	4.415 0.011 1.023, 3.808
Adequate	18.609 0.001 1.501, 4.934	8.772 0.001 5.816, 7.727	15.907 0.001 10.785, 12.030	11.474 0.001 7.237, 10.710
**Marital status** (Married vs. single)	3.801 0.292 -3.291, 1.894	4.620 0.002 4.664, 7.575	4.965 0.050 -0.007, 9.937	3.456 0.099 -0.656, 2.568
**Comorbidity** (no vs. yes)	8.141 0.001 3.712, 5.569	0.959 0.306 -2.804, 0.885	5.868 0.001 2.763, 4.972	3.231 0.014 0.664, 5.799
**Self-rated healthy** (vs. not healthy) Like others	1.211 0.062 -0.065, 2.345	2.406 0.099 -0. 576, 2.389	0.500 0.391 -0.625, 2.425	0.751 0.550 -1.899, 4.734
Better than others	10.016 0.001 2.115, 4.330	6.324 0.001 4.427, 6.730	9.620 0.001 8.840, 10.401	6.424 0.001 8.031, 11.690
**Living status** (alone vs. family)	-3.612 0.411 -5.044, 2.268	-3.081 0.094 -6.687, 0.525	-4.462 0.148 -1.605, 2.530	2.230 0.381 -2.787, 7.249
**R-squared**	0.400	0.378	0.456	0.339

Multivariate linear regression performed by STATA

^a^Coef

^b^*p*-value

^c^95% CI

Although there are many studies on the relationship between SWB and disease, there is room for further research because of the repercussions of various diseases. The elderly are a heterogeneous group in society, while many of them have adapted to the discomfort caused by the Covid-19, some senior citizens have suffered from a mental crisis during this period, which has severely affected their wellbeing. Therefore, taking measures to improve their well-being and investigate the factors involved in it can improve their QoL. The analysis of factors related to SWB in the elderly allows the necessary interventions to reduce the impact of these factors and help improve the perceived well-being of this age group. Moreover, the knowledge generated in this study helps to ensure the health, well-being and equality of this age group by formulating public policies in this field and emphasizing the preparation of human resources according to the needs of the elderly.

Well-being is a subjective construct, and different people are likely to evaluate different objective conditions differently depending on their goals, values, and even culture. For example, people in individualistic societies tend to focus on their own living conditions. In contrast, people in collectivist societies tend to consider the well-being of their families when evaluating their subjective well-being. On the other hand, the experience of old age also differs between societies. Therefore, for a better understanding of well-being in relation to health and illness, as well as the factors affecting it, more studies are needed in the form of longitudinal studies with different tools. However, trying to achieve this is a multidisciplinary approach that includes all fields of psychological, social, behavioral and brain sciences.

This study has limitations because SWB is a subjective assessment that relies on the perception, mood and attitude of the elderly, which changes over time. However, to reduce this limitation, we completed the questionnaire through an interview by an experienced person. Many potentially relevant variables including perceived stress, anxiety, depression and physical activity were not included in the analysis. The strengths of the study should also be mentioned. We were able to adjust age-related, potentially disruptive factors, including comorbidity, living arrangement and self-rated healthy. This study also showed a causal relationship between SWB and Covid-19 in the form of a case–control study, while most studies in the field of well-being and disease are based on cross-sectional data and have not addressed causation.

## Conclusions

This study provides evidence for the predictive effect of SWB on the risk of the Covid-19. Improving SWB at older ages may expand senior citizens’ longevity and enhance their good health. To promote SWB, we need to focus on the elderly with higher financial worries and comorbidities, as well as those with less education, health perception and SWB. Delaying a disability or chronic disease, in turn, can have consequences for health care costs, as fewer older people request hospital and primary care services. Therefore, it will be important for the elderly to determine strategies to improve SWB during the epidemic. It should also be noted that although SWB is important, it is only one component of health. Further research on a range of health indicators are needed to monitor and address the consequences of COVID-19.

## Supplementary Information

**Additional file 1:** **Table 1.** Associations between personal characteristics with emotional well-being and its subscales in elderly people. **Table 2.** Associations between personal characteristics with psychological well-being and its subscales in elderly people. **Table 3.** Associations between personal characteristics with social well-being and its subscales in elderly people.

## Data Availability

The data set used and / or analyzed of the study is available upon reasonable request from the corresponding author.

## Abbreviations

STROBE: STrengthening the Reporting of OBservational studies in Epidemiology
SWB: Subjective well-being
QoL: Quality of Life
CVD: Cardio Vascular Disease
OR: Odd Ratio
