# Supplementary material for: Subjective well-being predicts Covid-19 risk in the elderly: a case–control study
Source: BMC Geriatr. 2022 Nov 22;22:887. doi: 10.1186/s12877-022-03614-2 (PMC9682847; doi:10.1186/s12877-022-03614-2)
Supplement: Supplementary file 1 — Additional file 1: Table 1. Associations between personal characteristics with emotional well-being and its subscales in elderly people. Table 2. Associations between personal characteristics with psychological well-being and its subscales in elderly people. Table 3. Associations between personal characteristics with social well-being and its subscales in elderly people. [file 12877_2022_3614_MOESM1_ESM.docx]

**Table 1.** Associations between personal characteristics with emotional well-being and its subscales in elderly people

| **Determinants** | **Positive emotions** | **Negative emotions** | **Emotional well-being** |
| --- | --- | --- | --- |
| **Group**  (unhealthy vs. healthy) | -2.961  0.001  -4.050, -1.872 | 1.933  0.001  1.895, 2.970 | -4.894  0.001  -6.664, -3.125 |
| **Age**  Mean (SD) | -0.051  0.241  -0.137, 0.034 | 0.083  0.031  0.007, 0.159 | 0.032  0.646  -0.107, 0.172 |
| **Gender**  (female vs.male) | -0.677  0.304  -1.973,0.618 | 0.143  0.805  -1.002, 1.288 | -0.534  0.617  -2.640, 1.572 |
| **Occupation (**employed vs. unemployed) | 1.039  0.148  -0.373, 2.452 | -0.160  0.800  -1.409, 1.088 | 0.878  0.451  -1.418, 3.175 |
| **Education** (vs.illiterate**)**  **<** Diploma  > Diplomma | 0.728  0.243  -0.497, 1.954 | 1.487  0.007  0.404, 2.571 | -2.216  0.030  -5.877, -4.131 |
|  | -2.758  0.009  -2.214, -0.904 | 1.190  0.196  -0.620, 3.001 | -3.949  0.020  -3.100, -0.492 |
| **Econommic status** (vs. inadequate)  Almost enough  Adequate | 1.457  0.057  0.042, 2.957 | -1.728  0.011  -3.054, -0.403 | 3.186  0.011  2.623, 5.748 |
|  | 4.004  0.001  2.877, 5.131 | -4.767  0.001  -6.422, -3.111 | 8.772  0.001  5.816, 7.727 |
| **Marital status**  (Married vs single) | 2.822  0.003  1.003, 4.640 | -1.797  0.029  -3.404, -1.190 | 4.620  0.002  4.664, 7.575 |
| **Comoridity** (no vs. yes) | 0.035  0.951  -1.170, 1.099 | -0.923  0.071  -1.926, 0.079 | 0.959  0.306  -2.804, 0.885 |
| **Living status** (alone vs. family) | -1.535  0.174  -3.754, 0.683 | 1.545  0.122  -3.507, 0.415 | -3.081  0.094  -6.687, 0.525 |
| **R-squared** | 0.339 | 0.338 | 0.378 |

1. Coef.

2. p-value

3. 95% CI

**Table 2.** Associations between personal characteristics with psychological well-being and its subscales in elderly people

| **Determinants** | **Self-acceptance** | **Purpose in life** | **Environmental mastery** | **Positive relations with others** | **Personal growth** | **Autonomy** | **Psychological**  **Well-being** |
| --- | --- | --- | --- | --- | --- | --- | --- |
| **Group**  (unhealthy vs. healthy) | 0.319  0.462  -0.536, 1.175 | 0.0765  0.823  -0.597, 0.750 | -0.148  0.717  -0.954, 0.658 | 0.196  0.637  -0.624, 1.018 | 0.757  0.088  -0.114, 1.629 | -0.407  0.359  -1.282, 0.467 | 0.794  0.599  -2.182, 3.771 |
| **Age**  Mean (SD) | -.027  0.428  -0.094, 0.040 | 0.092  0.001  0.039, 0.145 | 0.133  0.001  0.069, 0.197 | -0.000  0.997  -0.064, 0 .064 | 0.139  0.001  0 .070, 0.207 | -0.001  0.965  -0.070, 0.067 | 0.336  0.005  0.101, 0.571 |
| **Gender**  (female vs.male) | 0.177  0.732  -0.841, 1.195 | 0 .266  0.514  -0.536, 1.068 | -0.115  0.812  -1.075, 0.843 | -0.988  0.048  -1.966, -0.010 | 0.119  0.821  -0.917, 1.156 | 0.572  0.279  -0.468, 1.613 | 0.030  0.986  -3.513, 3.574 |
| **Occupation(**employed vs. unemployed) | 0. .331  0.557  -0.779, 1.442 | -0.204  0.645  -1.080, 0.670 | 1.015  0.057  -0.030, 2.062 | 0.494  0.362  -0.572, 1.560 | 0.596  0.299  -0.534, 1.728 | 0.828  0.151  -0.306, 1.963 | 3.062  0.120  -0.802, 6.927 |
| **Education** (vs.illiterate**)**  **<** Diploma  > Diplomma | -0.796  0.105  -1.760, 0.167 | -0.450  0.244  -1.209, 0.309 | -1.514  0.001  -2.422, -0.606 | -1.042  0.027  -1.968, -0.117 | -1.300  0.010  -2.281, -0.318 | -0.269  0.589  -1.254, 0.714 | -5.374  0.002  -8.727, -2.020 |
|  | -1.619  0.049  -3.229, -0.008 | -1.131  0.080  -2.400, 0.137 | -1.217  0.115  -2.735, 0.299 | -0.023  0.976  -1.570, 1.522 | -1.982  0.018  -3.622, -0.342 | 0.377  0.652  -1.268, 2.022 | -5.598  0.050  -11.201, 0.005 |
| **Econommic status** (vs. inadequate)  Almost enough  Adequate | 1.167  0.052  -0.011, 2.346 | 0.384  0.414  -0.544, 1.313 | 1.323  0.020  0.212, 2.433 | 1.301  0.025  0.169, 2.432 | 1.178  0.054  -0.022, 2.379 | 0.372  0.542  -0.832, 1.577 | 5.727  0.006  1.626, 9.829 |
|  | 3.909  0.000  2.437, 5.382 | 0.918  0.060  -1.099, 1.221 | 3.321  0.000  1.933, 4.708 | 2.844  0.001  1.430, 4.258 | 3.203  0.001  1.703, 4.702 | 2.567  0.001  1.063, 4.153 | 15.907  0.001  10.785, 12.030 |
| **Marital status**  (Married vs single) | 0.816  0.261  -0.612, 2.246 | 0.585  0.306  -0.540, 1.711 | 1.139  0.097  -0.206, 2.486 | 0.773  0.268  -0.598, 2.145 | 1.518  0.041  0.063, 2.974 | 0.131  0.859  -1.329, 1.591 | 4.965  0.050  -0.007, 9.937 |
| **Comoridity** (no vs. yes) | 1.262  0.006  0.369, 2.154 | 0.203  0.569  -0.499, 0.906 | 1.427  0.001  0.586, 2.268 | 0.802  0.066  -0.053, 1.659 | 1.610  0.001  0.701, 2.519 | 0.562  0.225  -0.349, 1.474 | 5.868  0.001  2.763, 4.972 |
| **Living status** (alone vs. family) | 0.612  0.489  -1.131, 2.356 | -0.784  0.261  -2.159, 0 .589 | -1.755  0.036  -3.399, -0.112 | -0.701  0.409  -2.376, 0 .972 | -2.480  0.006  -4.256, -0.704 | 1.100  0.225  -0.682, 2.882 | -4.462  0.148  -1.605, 2.530 |
| **R-squared** | 0.262 | 0.181 | 0.412 | 0.224 | 0.401 | 0.157 | 0.456 |

1. Coef.

2. p-value

3. 95% CI

**Table 3.** Associations between personal characteristics with social well-being and its subscales in elderly people

| **Determinants** | **Social coherence** | **Social integration** | **Social acceptance** | **Social contribution** | **Social actualization** | **social well-being** |
| --- | --- | --- | --- | --- | --- | --- |
| **Group**  (unhealthy vs. healthy) | -0.998  0.037  -1.938, -0.058 | -0.597  0.213  -1.540, 0.345 | 0.248  0.522  -0.516, 1.014 | -0.883  0.002  -2.230, -1.058 | -1.544  0.001  -2.244, -0.844 | -2.007  0.109  -4.470, 0.454 |
| **Age**  Mean (SD) | 0.011  0.765  -0.062, 0.085 | -0.009  0.801  -0.083, 0.064 | -0.011  0.701  -0.072, 0.048 | 0.021  0.324  -0.021, 0.065 | -0.051  0.065  -0.107, 0.003 | -0.040  0.684  -0.234, 0.154 |
| **Gender**  (female vs.male) | 0.506  0.372  -0.611, 1.625 | -.705  0.216  -1.828, 0.416 | -.277  0.548  -1.189, 0.633 | 1.407  0.001  0.749, 2.064 | 0.050  0.904  -0.781, 0.883 | 0.981  0.510  -1.949, 3.912 |
| **Occupation (**employed vs. unemployed) | 0.695  0.262  -.523, 1.915 | 1.394  0.026  0.171, 2.618 | 0.558  0.269  -0.435, 1.551 | 0.866  0.018  0.148, 1.583 | 0.745  0.107  -0.162, 1.654 | 2.529  0.120  -0.667, 0.725 |
| **Education** (vs.illiterate**)**  **<** Diploma  > Diplomma | -1.842  0.001  -2.900, -0.784 | -0.994  0.066  -2.056, 0.067 | 0.313  0.474  -0.548, 1.175 | -1.029  0.001  -1.651, -0.407 | -0.006  0.987  -0.794, 0.781 | -3.559  0.012  -6.333, -0.786 |
|  | -1.728  0.055  -3.497, 0.039 | -0.760  0.399  -2.534, 1.014 | -1.686  0.022  -1.801, -0.704 | -1.826  0.001  -2.866, -0.786 | 0.149  0.823  -1.167, 1.465 | -2.480  0.292  -7.114, 2.153 |
| **Econommic status** (vs. inadequate)  Almost enough  Adequate | 1.236  0.061  -0.057, 2.530 | 0.668  0.311  -0.629, 1.967 | -0.141  0.791  -1.196, 0.912 | 1.252  0.001  0.491, 2.013 | 1.400  0.005  0.436, 2.364 | 4.415  0.011  1.023, 3.808 |
|  | 2.407  0.004  0.791, 4.023 | 3.829  0.000  2.207, 5.451 | 0.635  0.342  -0.681, 1.952 | 1.626  0.001  0.675, 2.577 | 2.975  0.001  1.771, 4.179 | 11.474  0.001  7.237, 10.710 |
| **Marital status**  (Married vs single) | 1.250  0.117  -0.318, 2.819 | .763  0.340  -0.811, 2.338 | 1.215  0.062  -0.062, 2.493 | 0.501  0.285  -.420, 1.424 | -0.275  0.643  -1.443, 0.893 | 3.456  0.099  -0.656, 2.568 |
| **Comoridity** (no vs. yes) | 1.831  0.001  0.852, 2.811 | 0.969  0.053  -0.013, 1.953 | -0.454  0.263  -1.252, 0.343 | -0.114  0.694  -0.690, 0.461 | 0.999  0.008  0.269, 1.729 | 3.231  0.014  0.664, 5.799 |
| **Living status** (alone vs. family) | 0.690  0.478  -1.224, 2.604 | 0.477  0.625  -1.444 , 2.398 | -1.789  0.025  -3.349, -0.228 | 0.005  0.993  -1.120, 1.131 | -0.730  0.313  -2.156 , 0.695 | 2.230  0.381  -2.787, 7.249 |
| **R-squared** | 0.328 | 0.276 | 0.096 | 0.357 | 0.319 | 0.339 |

Multivariate linear regression performed by STATA

1. Coef.

2. p-value

3. 95% CI
